# Who is Looking After Mom and Dad? Unregulated Workers in Canadian Long-Term Care Homes*

**DOI:** 10.1017/S0714980814000506

**Published:** 2015-03

**Authors:** Carole A. Estabrooks, Janet E. Squires, Heather L. Carleton, Greta G. Cummings, Peter G. Norton

**Affiliations:** 1 Faculty of Nursing, University of Alberta; 2 Ottawa Hospital Research Institute and School of Nursing, University of Ottawa; 3 Department of Family Medicine, University of Calgary

**Keywords:** aging, care aides, long-term care, nursing homes, personal care workers, unregulated healthcare workforce, vieillissment, aides de soins, soins a longue durée, main d'oeuvre de services de santé non-règlementé

## Abstract

Older adults living in residential long-term care or nursing homes have increasingly complex needs, including more dementia than in the past, yet we know little about the unregulated workforce providing care. We surveyed 1,381 care aides in a representative sample of 30 urban nursing homes in the three Canadian Prairie provinces and report demographic, health and well-being, and work-related characteristics. Over 50 per cent of respondents were not born in Canada and did not speak English as their first language. They reported moderately high levels of burnout and a strong sense of their work’s worth. Few respondents reported attending educational sessions. This direct caregiver workforce is poorly understood, has limited training or standards for minimum education, and training varies widely across provinces. Workplace characteristics affecting care aides reflect factors that precipitate burnout in allied health professions, with implications for quality of care, staff health, and staff retention.

## RÉSUMÉ

Les personnes âgées recevant soins de longue durée ou soins infirmiers résidentiels ont des besoins de plus en plus complexes, y compris exhibitant plus de la démence que dans le passé, mais on ne connait que peu en ce qui concerne la population non réglementée qui fournit leurs soins. Nous avons interrogé 1 381 aides dans un échantillon représentatif de 30 maisons de soins infirmiers en milieu urbain dans les trois provinces des Prairies canadiennes afin de signaler des caractéristiques démographiques, la santé et le bien-être, et des caractéristiques liées au travail. Plus de 50 pour cent des répondants ne sont pas nés au Canada et ne parlaient pas l'anglais comme leur première langue. Ils ont signalé des niveaux d'épuisement modérément élevés et un sentiment fort de la valeur de leur travail. Peu de répondants ont déclaré avoir participé à des séances de formation. Cette main d'oeuvre composée de fournisseurs de soins directs est mal comprise, leur formation et les normes pour l'emploi sont limitées, et la formation varie considérablement d'une province à l'autre. Les caractéristiques du lieu de travail qui touchent aux aidants reflètent des facteurs qui favorisent l'épuisement professionnel dans les professions connexes de la santé, avec des implications pour la qualité des soins, pour la santé du personnel et pour leur rétention.

This article describes the workforce providing the majority of direct care to the frail elderly in nursing homes. We report on health, well-being, job and continuing education characteristics of this workforce and discuss policy and management implications that arise. The current lack of information about the numbers and characteristics of this occupational group reduces our ability to undertake effective workforce planning and to monitor progress toward achieving workforce improvements that result in acceptable quality of care. Our research question on the current characteristics of the care aide workforce in long term care (LTC) homes illuminates effective routes to modify those characteristics. This analysis highlights factors that, if modified, could significantly improve quality of care and quality of life for LTC residents, and quality of work life for care aides.

By 2036, the number of Canadians 65 and older will more than double to 10.4 million, with growth most rapid in those over 75 years of age (Statistics Canada, 2010). After age 65, age-related dementias (ARDs) and other neurodegenerative diseases begin to take their greatest toll on quality of life and produce the greatest proportional costs to the health system. Presently, one in 40 Canadians aged 65–74 and one in three Canadians over age 85 have ARD (Canadian Institutes of Health Research, 2010). By 2038, 1.125 million Canadians, or 2.8 per cent of the population, are projected to have an ARD (Alzheimer Society of Canada, 2010). Recent prevalence figures in the United States are higher (Alzheimer’s Association, 2013). In the United States, 70 per cent of people with dementia die in a nursing home (Mitchell, Teno, Miller, & Mor, 2005); in Europe, this figure ranges from 50 per cent (Wales) to 92 per cent (Netherlands) (Houttekier et al., 2010). As the number of older adults with dementia increases, so will the need for supportive living and residential long-term care, without dramatic breakthroughs in either prevention or treatment of ARD. Recent Canadian projections estimate that the need for LTC beds will rise 10-fold by 2038 (Alzheimer Society of Canada).

In addition to being in greater demand, residential LTC facilities (nursing homes) in Canada have become more complex care environments (Hirdes, Mitchell, Maxwell, & White, 2011). Increasingly, older adults are admitted later in the trajectories of their dementia and other chronic diseases and are thus more dependent, with more-complex needs and in a greater state of vulnerability. Few new residents to long-term care have low care needs (Ikegami, Morris, & Fries, 1997; Mor et al., 2007) while 60 per cent have significant and often co-morbid care challenges (Doupe et al., 2012); more than 70 per cent have an ARD (Doupe et al., 2011; Gruber-Baldini et al., 2009). These residents with their advancing age, loss of family and other supports, and their severe communication difficulties are among our most vulnerable and at-risk citizens.

Unregulated, non-professional workers (care aides) provide 75 to 80 per cent of direct care to nursing home residents in Canada (Janes, Sidani, Cott, & Rappolt, 2008; Kontos, Miller, Mitchell, & Cott, 2011). Staff levels and skill sets have not kept pace with resident acuity (Feng, Grabowski, Intrator, Zinn, & More, 2008; McGregor et al., 2010); moreover, nursing home care providers report increased workloads and decreased quality of work life (Bostick, 2004). The high prevalence of ARD in nursing homes is associated with increased care provider job strain (Morgan, Semchuk, Stewart, & D’Arcy, 2002), reduced job satisfaction (McGilton, McGillis Hall, Wodchis, & Petroz, 2007), and increased staff turnover (Bostick, Rantz, Flesner, & Riggs, 2006).

In the United States, the majority of care aides are female (86%), over 40 years of age, born in the United States (77%), earn less than half the US national median annual earnings, and have a high school education or less (55%) (Fredriksen-Goldsen & Bonifas, 2013; PHI National, 2011; Potter, Churilla, & Smith, 2006). Less is known about the Canadian care aide workforce. We were unable to identify a single comprehensive study or report that focused on this workforce and associated demographic characteristics. Individual Canadian studies report that these workers are predominantly women (> 90%), and on average are just over 46 years old (Morgan, Stewart, D’Arcy, Forbes, & Lawson, 2005). About half are Canadian-born (Chappell & Novak, 1992), two-thirds speak English as their native language (McGilton et al., 2007), and in 2003, 63 per cent held a post-secondary certificate or diploma (Statistics Canada, 2004).

In Canada, there is no national education standard for care aides to enter practice (Berta, Laporte, Deber, Baumann, & Gamble, 2013; Canadian Nurses Association, 2005; Pyper, 2004), although most jurisdictions require formal college-level training for new workers (Canadian Nurses Association). Provincial care aide program curricula are offered in six provinces (British Columbia, Alberta, Ontario, Quebec, Newfoundland and Labrador, and Nova Scotia) while others use institutionally “approved” curricula (Association of Canadian Community Colleges [ACCC], 2012) resulting in significant variation in the level of preparation offered by providers across the provinces (Berta et al., 2013). For example, an environmental scan of care aide programs across Canada reported programs as short as 19 weeks (531 contact hours) in Alberta to over 34 weeks (795 contact hours) in the Yukon (ACCC). Additionally, because they are unregulated workers, in Canada no regulatory or other body monitors or follows the labour supply of this workforce. This has been reported as a problem worldwide (OECD, 2013), and only recently has the United States been able to release its first comprehensive analysis of the labour supply of direct care workers (Baughman & Smith, 2012).

In Canada, some provinces are developing registries of workers. In 2010, British Columbia began mandatory registration of care aides in publicly funded nursing homes (BC Care Aide and Community Health Worker Registry, 2013); in 2010, Nova Scotia began voluntary registration (Health Association Nova Scotia, 2012); in 2011, Alberta initiated an employer-implemented directory of care aides; and in 2012, Ontario established registration for home care workers with future phases to include workers in residential LTC facilities (PSW Registry Ontario, 2013) (see Table 1 for more detailed information). With a potentially vulnerable and variably trained workforce providing the majority of direct care for a group of frail, older Canadians with highly complex care and dependency needs, quality of care is a significant concern. For 40 years, reports at the international (OECD, 2005; Tolson et al., 2011), national (Baum, 1977; Institute of Medicine, 2001; Moss & Halamandaris, 1977; National Advisory Council on Aging, 2005; Vladeck, 1980), and provincial (British Columbia Office of the Ombudsperson, 2009, 2012; Dunn, 2005; Hyde, 1981; Long-Term Care Task Force Ontario, 2012) levels have highlighted serious concerns for quality of care in nursing homes.

**Table 1:** Existing care aide registries by Canadian province (All registries = acute care, assisted living, long-term care, home care, community care settings)

Registry characteristics	BC	AB	ON	QC	NS
Is registry mandatory?^a^	Yes	No^b^	Yes	No	No
Setting	All	All	Home Care^c^	All	All
Annual registration fee (for care aide)	Nil	Nil	Nil	$100^d^	$57.50^e^
Minimum education requirements to self-register?^f^	Yes	NA^g^	Yes	Yes	Yes
**Data within the Registry**					
Demographic and contact information	Yes	Yes	Yes	Yes	Yes
Workforce employment, attrition, and mobility information	Yes	Yes	Yes	Yes	Yes
Educational background/certification	Yes	Yes	Yes	Yes	Yes
**Benefits of Registry**					
Online visibility to employers^h^	Yes	No	Yes	Yes	Yes
Employer can verify registration	Yes	NA	Yes	No	Yes
Public availability (i.e., public can verify registration)	No	No	No^i^	No	Yes
Tracks alleged incidences of abuse and has a process for termination	Yes^j^	No	No	No	No
Mails or posts professional development opportunities	Yes	No	No	Yes	Yes
Collective/group insurance plan	No	No	No	Yes^k^	No

BC = British Columbia

AB = Alberta

ON = Ontario

QC = Quebec

NS = Nova Scotia

a Mandatory only for care aides working in publicly funded health-care organizations.

b No = Alberta has a “directory” of care aides. It is the responsibility of the employer to submit information to the directory with regards to those they employ as a care aide, but it is not legislated or included in contracts with service providers.

c Home Care is the first setting Ontario is pursuing with its newly developed (2012) registry. The Government of Ontario claims to be moving forward on a mandatory registration policy for all publicly funded care aides (PSW Registry Ontario (http://www.pswregistry.org/), although no target dates are reported.

d Retrieved 28 August 2013 from personal communication with president of the Provincial Association of Orderlies and Patient Care Attendants (FPBQ).

e $57.50 is the annual registration fee NS care aides pay, which was implemented in 2013. There was no fee for the first two years the registry opened. (Continuing Care Assistant Program: CCA Registry, Retrieved 20 August 2013 from http://www.novascotiacca.ca/).

f Minimum education requirements to self-register are only for new workers and comprise completion of an “approved” program, which varies greatly by province. Only in Nova Scotia are care aides required to pass a standardized certification exam.

g Not applicable because care aides in Alberta cannot self-register. They are entered into the directory only if they are both employed and their employer enters them into the directory.

h BC and NS registries enable care aides to post online profiles for employers to seek them out while ON’s registry website has claims that a similar function will be available in the future. ON’s registry currently has an online job board for care aides to search (http://www.pswregistry.org/).

i ON’s registry website has claims that this function will be available in the future.

j Retrieved 20 August 2013 from http://www.cachwr.bc.ca/.

k Retrieved 28 August 2013 from personal communication with president of the Provincial Association of Orderlies and Patient Care Attendants (FPBQ).

In this study, we answered the research question: What are the demographics and select health and work-related outcomes of the unregulated workforce in western Canadian nursing homes? We were also interested in whether these demographic and other characteristics differed by province or owner-operator model in our sample.

## Methods

All data were collected within the *Translating Research in Elder Care* (TREC) study, a five-year research program seeking to identify modifiable features of organizational context that are associated with better resident and staff outcomes in LTC home settings (Estabrooks, Hutchinson, et al., 2009; Estabrooks, Squires, Cummings, Teare, & Norton, 2009). The breadth and depth of the TREC data collection makes possible secondary studies such as ours that ask a defined research question about a select subset of the TREC data.

### Sampling and Measures (Nursing Homes)

TREC is situated in 36 nursing homes (30 urban, six rural) in Alberta, Saskatchewan, and Manitoba. All nursing homes that met the TREC inclusion criteria were eligible to participate (Estabrooks et al., 2009). The TREC team selected nursing homes using stratified (health region, owner-operator model, size) random sampling in order to ensure representation of these dimensions in our sample. For this study, we used data from the 30 urban LTC homes. LTC homes fell into three categories of owner-operator models: (1) public – a facility supported primarily through public funds, owned and operated by the local government; (2) voluntary – a facility run by a voluntary, cultural, or religious organization; and (3) private (for profit) – a facility in which the individuals or agency in control receive compensation (other than wages, rent, or other expenses) for the services they provide. Our sample of urban LTC homes included 22 small homes (35 to149 beds) and 8 large homes (≥ 150 beds). We report here on data collected in 2009–2010.

### Sampling and Data Collection (Care Aides)

Care aides completed the TREC survey, a suite of instruments designed to measure (a) demographic characteristics, (b) organizational context, (c) use of best practices, (d) staff outcomes, and (e) factors believed to influence the use of best practices. To sample, we used a census of eligible care aides. All care aides who met the inclusion criteria (see Table 2) were invited to participate in the survey, and we collected data from all who accepted the invitation (Estabrooks et al., 2009). Trained data collectors administered the survey to care aides using computer-assisted, structured personal interviews (Squires et al., 2012). Care managers and facility administrators completed unit and facility surveys respectively.

**Table 2:** Inclusion criteria (care aides)

Inclusion	Exclusion
• Have worked for at least three months and are working now	• Health care aide student
• Work a minimum of six shifts per month on this unit	

### Care Aide Measures

In this article, we report on the following variables collected in our study from care aides: demographics, work-related variables, health status and burnout, and continuing education.

#### Work-Related Variables

We measured (a) job and vocational satisfaction, and (b) whether care aides had enough orientation and job knowledge. We did so by using single items scored on a five-point Likert agreement scale ranging from strongly disagree (1) to strongly agree (5). These single items have produced consistent findings in our previous pilot work with care aides (Boström, Squires, Mitchell, Sales, & Estabrooks, 2012) and past studies with nurses (Estabrooks, Squires, Adachi, Kong, & Norton, 2008; Squires et al., 2013) indicating reliability.

#### Dementia-Related Responsive Behaviours

We measured these behaviours, exhibited towards staff by residents, by using six items (threat of assault, emotional abuse, physical abuse, verbal sexual harassments, sexual assault, and forced sexual intercourse). Each item was scored as yes or no.

#### Health Status

The SF-8™ health survey (Ware, Kosinski, Dewey, & Gandek, 2001) assesses mental and physical health status using eight items. The eight items have been selected from pools of empirically tested items, and scored on the same norm-based metric as the original larger SF-36^®^ scale (Carr, 2003). Responses were on a five- or six-point scale. Scoring was done using a proprietary algorithm obtained when permission to use the scale was granted us.

#### Burnout

The Maslach Burnout Inventory General Survey (MBI-GS) (Maslach & Jackson, 1981; Maslach, Jackson, & Leiter, 1996) has three subscales (emotional exhaustion, cynicism, job efficacy). The original MBI-GS contained 16 items. In this study, we used the MBI-GS (short form), which consists of nine items (three items for each of the three subscales, each scored on a seven-point Likert scale). The mean was taken for each subscale. A low risk for burnout is reflected by one or more of the following: emotional exhaustion score of < 1.67, cynicism < 1.00, and efficacy > 4.00. A high risk for burnout is reflected by one or more of the following: emotional exhaustion > 3.00, cynicism > 2.33, and efficacy < 3.30.

#### Continuing Education

Care aides were asked how often they attended in-services, workshops, or courses in the past year, and managers were asked if there were one or more clinical educators in the facility.

Additional details on each of these variables are presented in Additional File 1.

### Statistical Analyses

We calculated means and standard deviations for interval data and frequency counts and proportions for categorical data. To assess differences between provinces, owner-operator models (private, public, voluntary), and facility size (small, medium, large) on categorical and interval-level variables, we used contingency chi-square tests and one-way ANOVA respectively with a post-hoc test (Bonferroni correction for interval-level variables; e.g., years worked as a care aide, and binary or multinomial logistic regression for categorical variables; e.g., age). To assess for a possible province effect, we calculated statistical significance (using ANOVA *p* value and effect size) of all work-related and staff outcome variables before and after adjusting for three factors: sex, education (care aide certificate), and whether the care aide was born in Canada.

We were also interested to observe if any one of these three sampling dimensions (province, owner-operator model, facility size) were more strongly associated with the three educational and/or professional development opportunities for care aides that were available to us. To determine this possible association, we conducted two-level individual logistic regressions (one model for having a care aide certificate and one for in-services attended) using each of our three educational variables as dependent variables) that controlled for the three sampling dimensions. We compared these same sampling dimensions using the presence of a clinical educator (facility level) with Fisher’s exact test.

### Ethics

Ethics approvals were obtained from the research ethics boards of all investigator-affiliated universities. Operational approvals were obtained from participating organizations.

## Results

### Sample Characteristics

A total of 1,381 health care aides (representing approximately 70% of those eligible to participate during the period in which we collected data in each facility) completed the TREC survey in year two (July 2009–June 2010) (see Table 3). The majority (*n* = 837, 60.6%) were from Alberta, followed by Manitoba (*n* = 336, 24.3%) and Saskatchewan (*n* = 208, 15.1%). Many worked primarily day shifts (665, 48.2%), with 548 (39.7%) working evening shifts and 168 (12.2%) working night shifts. Just over half of the sample reported speaking English as their first language. We found statistically significant differences between provinces on all demographic characteristics with the exception of age and whether the care aide had completed a high school diploma. Select (but fewer) demographic variables also differed by owner-operator model (e.g., being born in Canada, hours worked in two weeks, and years on unit) and facility size (sex, first language spoken, and being born in Canada) (See Additional File 2).

**Table 3:** Demographic characteristics of care aide sample

Variables		Province				χ^2^ / ANOVA	
		Alberta (AB) *n = 837*	Saskatchewan (SK) *n = 208*	Manitoba (MB) *n = 336*	Total *n = 1381*	*p* value^a^	post-hoc^b^
**Age** [*n*, (%)]	< 20 years	9 (1.1)	1 (0.5)	2 (0.6)	12 (0.9)	.584	N/A
	20–29 years	105 (12.5)	31 (15.0)	30 (8.9)	166 (12.0)		
	30–39 years	177 (21.1)	46 (22.2)	78 (23.2)	301 (21.8)		
	40–49 years	267 (31.9)	65 (31.4)	103 (30.7)	435 (31.5)		
	50–59 years	218 (26.0)	46 (22.2)	94 (28.0)	358 (25.9)		
	> 60 years	61 (7.3)	18 (8.7)	29 (8.6)	108 (7.8)		
**Sex** [*n*, (%)]	Male	60 (7.2)	6 (2.9)	37 (11.0)	103 (7.5)	.002	a, b, c
	Female	776 (92.8)	202 (97.1)	298 (89.0)	1276 (92.5)		
**Shift worked most of the time** [*n*, (%)]	Day Shift	394 (47.1)	106 (51.0)	165 (49.1)	665 (48.2)	.014	a, b
	Evening Shift	355 (42.4)	78 (37.5)	115 (34.2)	548 (39.7)		b
	Night Shift	88 (10.5)	24 (11.5)	56 (16.7)	168 (12.2)		a, b
**First language** [*n*, (%)]	English	408 (48.8)	160 (77.3)	138 (41.1)	706 (51.2)	< .001	a, c
	Filipino	61 (7.3)	10 (4.8)	62 (18.5)	133 (9.6)		b, c
	Tagalog	165 (19.7)	10 (4.8)	73 (21.7)	248 (18.0)		a, c
	Other	202 (24.2)	27 (13.0)	63 (18.8)	292 (21.2)		a, c
**Born in Canada** [*n*, (%)]		299 (35.7)	158 (76.0)	93 (27.7)	550 (39.8)	< .001	a, b, c
**Hours worked in 2 weeks** [Mean, (*SD*)]		61.294 (17.850)	66.082 (16.178)	65.535 (17.582)	63.047 (17.666)	< .001	a, b
**Years worked as care aide** [Mean, (*SD*)]		9.788 (8.705)	11.782 (9.209)	11.890 (8.557)	10.600 (8.799)	< .001	a, b
**Years worked in unit** [Mean, (*SD*)]		4.335 (4.922)	7.004 (7.423)	4.875 (5.477)	4.868 (5.575)	< .001	a, c

*SD* = standard deviation

a Chi-square test for categorical variables and one-way ANOVA for quantitative variables.

b The post-hoc test was examined using Bonferroni correction for continuous outcomes and (binary or multinomial) logistic regression for categorical outcomes. Letters *a*, *b*, and *c* denote the post-hoc test (multiple comparison) result for AB-SK, AB-MB, and SK-MB respectively (e.g., *a* implies that a difference exists between AB and SK).

### Work-Related Health and Burnout Characteristics

Mean scores by province for the work-related variables and health and well-being outcomes are presented in Table 4; mean scores by owner-operator model and facility size can be found in Additional File 2. The care aides overall reported being satisfied with their job and with being a care aide, and perceived that they had adequate knowledge and orientation to carry out their job. They reported experiencing, on average, three types of dementia-related responsive behaviours during the last five shifts worked. Mental and physical health subscale scores on the SF-8 health survey were 51.0 (*SD* = 8.7) and 49.4 (*SD* = 8.0) respectively. Care aides had moderate risk levels for burnout on two of the three burnout subscales: exhaustion and cynicism. On the third subscale, job efficacy, aides reported unusually high levels (higher is better).

**Table 4:** Comparison of work-related and health outcomes among care aides by province

Variables	Province							
	Alberta *n = 837*	Saskatchewan *n = 208*	Manitoba *n = 336*	Total *n = 1381*	ANOVA (unadjusted)		ANOVA (adjusted^a^)	
					*p* value	ES^b^	*p* value	ES
**Work Related [*n*, (%)]**								
Job satisfaction^c^	4.116 (.796)	3.832 (.941)	4.143 (.735)	4.080 (.812)	< .001	.017	.011	.036
Vocational satisfaction^c^	4.241 (.765)	4.053 (.902)	4.140 (.796)	4.188 (.797)	.004	.008	.012	.012
Adequate knowledge^c^	4.203 (.695)	3.894 (.921)	4.080 (.751)	4.127 (.754)	< .001	.022	< .001	.038
Adequate orientation^c^	4.129 (.805)	3.803 (.960)	4.158 (.701)	4.087 (.815)	< .001	.022	.004	.050
**Health Status [*n*, (%)]**								
Physical health status^d^	49.628 (8.097)	47.656 (8.533)	49.699 (7.259)	49.346 (7.996)	.004	.008	.169	.022
Mental health status^d^	51.571 (8.780)	48.357 (8.467)	51.226 (8.216)	50.998 (8.666)	< .001	.017	.013	.047
**Burnout [*n*, (%)]**								
MBI exhaustion-energy^e^	2.373 (1.631)	2.795 (1.624)	2.652 (1.572)	2.504 (1.624)	.001	.011	.001	.013
MBI cynicism-involvement^e^	2.049 (1.532)	2.468 (1.730)	2.428 (1.618)	2.203 (1.595)	< .001	.015	< .001	.020
MBI efficacy-inefficacy^e^	5.305 (.839)	4.945 (1.000)	5.196 (.885)	5.224 (.885)	< .001	.021	< .001	.035
Dementia-related responsive behaviours (aggression) towards staff^f^	3.038 (1.754)	3.284 (1.462)	3.256 (1.648)	3.128 (1.690)	.048	.004	.029	.008

ES = effect size

MBI = Maslach Burnout Inventory

a Province effect after adjusting for sex, education (care aide certificate), and whether born in Canada.

b Effect size (Cohen’s *f*^2^): small effect = 0.02, medium effect = 0.15, large effect = 0.35.

c Each variable was asked with a single item, scored on a five-point Likert scale (1 = strongly disagree to 5 = strongly agree).

d Physical and mental health status were measured using the Health Status Short Form (SF-8) which contains eight items. Responses are on a five- or six-point scale, and scoring is done using a proprietary algorithm obtained when permission to use the scale is granted. Higher scores indicate better perceived health status. These figures are an average across age groupings.

e Burnout was measured using the Maslach Burnout Inventory General Survey (MBI-GS), which consists of three subscales (emotional exhaustion, cynicism, job efficacy), each containing three items. All items are scored on a seven-point frequency Likert scale (0 = never to 6 = daily). A mean is calculated for each subscale. High scores on exhaustion and cynicism with low scores on efficacy indicate high risk for burnout.

f Dementia-related responsive behaviour towards staff is measured by asking care aides to report whether or not they have experienced six kinds of responsive behaviours by a resident in their last five shifts. A count of the kinds of responsive behaviours they indicated experiencing is taken for a total score between 0 and 6.

After adjusting for sex, education (care aide certificate), and whether or not the care aide was born in Canada, all variables except physical health status were significantly different (*p* < .05) between provinces (see Table 4). However, all effect sizes (less sensitive to large sample sizes) were small. To further assess whether a “true” province effect existed, we selected a five per cent random sample. Our sampling strategy ensured sufficient variation to permit inclusion of province, facility size, and owner-operator model as substantive predictor variables (see Table 4 and Table 5). Analysis on this sample resulted in only two variables (adequate knowledge and adequate orientation) displaying statistically significant differences between provinces. Only one variable (MBI Cynicism-Involvement) was statistically different between owner-operator model and facility size after adjusting for sex, education (care aide certificate), and whether or not the care aide was born in Canada (see Additional File 2). Cynicism was higher in private facilities than in voluntary or public facilities; cynicism was also higher in medium-sized facilities than in small or large facilities.

**Table 5:** Logistic regression results

(DV: Care aide certificate^a^)								
Variable (Co-variate)	Beta	*SE*	Wald	*df*	Sig.	Exp(Beta)	95% CI for Exp(Beta)	
							Lower	Upper
Province: AB^b^	–1.524	.266	32.890	1	.000	.218	.129	.367
Province: SK	–1.342	.281	22.736	1	.000	.261	.151	.454
Site size: Small^c^	–.642	.186	11.968	1	.001	.526	.366	.757
Site size: Medium	–.753	.215	12.276	1	.000	.471	.309	.718
O-O model: Public^d^	.577	.192	9.033	1	.003	1.780	1.222	2.593
O-O model: Private	–.466	.185	6.371	1	.012	.628	.437	.901
**(DV: Attend in-services/workshops/courses,**^e^ **Individual level data)**								
Province: AB	–.097	.165	.347	1	.556	.907	.657	1.253
Province: SK	–1.105	.206	28.764	1	.000	.331	.221	.496
Site size: Small	–1.499	.174	74.395	1	.000	.223	.159	.314
Site size: Medium	–.307	.152	4.072	1	.044	.735	.546	.991
O-O model: Public	.178	.148	1.446	1	.229	1.195	.894	1.599
O-O model: Private	.858	.153	31.533	1	.000	2.358	1.748	3.181

*df* = degrees of freedom

DV = dependent variable

O-O = owner-operator

*SE* = standard error

Sig. significance

a Probability (HCA certificate = Yes) was modelled

b Reference group = Manitoba (MB)

c Reference group = Large

d Reference group = Voluntary

e Probability (Attend in-services/workshops/courses = 1) was modelled

### Continuing Education

In Table 6, we report three variables related to educational opportunities for care aides. The majority (83.6%) of care aides reported having a care aide certificate while fewer than 50 per cent reported attending in-services, workshops, or courses regularly in the past year. Significant differences between provinces (see Table 6), owner-operator models (see Additional File 2), and facility size (see Additional file 2) were noted with respect to both variables: having a care aide certificate and attending in-service sessions regularly. In addition, most (73.3%) of the TREC nursing homes had a clinical educator; however, significant differences were present only between provinces (28.6% Saskatchewan, 86.7% Alberta, 87.5 % Manitoba).

**Table 6:** Educational opportunities for care aides by province

Individual Level Variable	Province, *n* (%)				χ^2^-test (*p* value)
	Alberta	Saskatchewan	Manitoba	Total	
	*n = 837*	*n = 208*	*n = 336*	*n = 1381*	
**Education** [*n*, (%)]					
High school	768 (92.1)	192 (92.3)	317 (94.3)	1,277 (92.7)	.397
Care aide certificate	680 (81.5)	161 (77.4)	311 (92.6)	1,152 (83.6)	< .001^a^
**Attend in-services/workshops/courses** [*n*, (%)]	413 (49.5)	54 (26.0)	191 (57.2)	658 (47.8)	< .001

Facility level variable	Province, *n* (%)				Fisher’s exact test^b^ (*p* value)
	Alberta	Saskatchewan	Manitoba	Total	
	*n = 15*	*n = 7*	*n = 8*	*n = 30*	
Clinical Educator (Yes)	**13 (86.7)**	**2 (28.6)**	**7 (87.5)**	**22 (73.3)**	.013

a *a*, *b*, and *c* denote the post-hoc test (multiple comparison) result for AB-SK, AB-MB, and SK-MB respectively (e.g., *a* implies that a difference exists between AB and SK).

b Fisher’s exact test was used because 50% of the cells have expected a count less than five.

Table 5 presents findings from the logistic regression models that we ran to determine which sampling dimensions of province, owner operator model, and facility size were more strongly associated with having a care aide certificate and attending in-services. Findings show that all three dimensions (province, owner operator model, facility size) were significant predictors of having a care aide certificate as well as attending in-services. Owner-operator model (public vs. voluntary) displayed the strongest beta coefficient in both models. Only province was a significant predictor of a facility’s having a clinical educator in place (see Table 7).

**Table 7:** Association between clinical educator and province, size, and owner-operator model (facility-level data)

Variable		Clinical educator		Total (*n* = 30)	Fisher’s exact test (*p* value)^a^
		No (*n* = 8)	Yes (*n* = 22)		
		[*n*, (%)]	[*n*, (%)]	[*n*, (%)]	
Province	Alberta	2 (25.0)	13 (59.1)	15 (50.0)	.013
	Saskatchewan	5 (62.5)	2 (9.1)	7 (23.3)	
	Manitoba	1 (12.5)	7 (31.8)	8 (26.7)	
Site size	Small	3 (37.5)	6 (27.3)	9 (30.0)	.180
	Medium	4 (50.0)	5 (22.7)	9 (30.0)	
	Large	1 (12.5)	11 (50.0)	12 (40.0)	
Owner-Operator model	Public	3 (37.5)	5 (22.7)	8 (26.7)	.866
	Private	2 (25.0)	6 (27.3)	8 (26.7)	
	Voluntary	3 (37.5)	11 (50.0)	14 (46.7)	

a Fisher’s exact test was used because 50% of the cells have expected count less than five.

## Discussion

We report the first data, to our knowledge, for Canadian nursing home care aides on demographic, health status, burnout, job and vocational satisfaction, and continuing educational opportunities. Care aides in Canadian prairie provinces tend to be middle-aged and older women, with a high school diploma; some have care aide certificate-level education. In urban centers, half of these workers were born outside of Canada and do not speak English as a first language. Care aides in this study report high levels of job and vocational satisfaction. They have mean scores for physical and mental health from the SF-8 survey consistent with U.S. general population norms (Ware, Kosinski, & Keller, 1996). Consistent with other Canadian reports (Boström et al., 2012; Morgan et al., 2005; Morgan et al., 2012), they report regularly experiencing dementia-related responsive behaviours from residents. They are at moderate risk of burnout, consistent with other reports of nurses’ burnout in the literature (Leiter & Maslach, 2009). However, their reported job efficacy – the sense that their work is meaningful and has purpose – is unusually high. This finding is consistent with discussions of intrinsic rewards in the direct care workforce by Morgan, Dill, and Kalleberg (2013) and Rose (2003).

These findings are similar to those reported in the U.S. studies and in several small Canadian studies. However, our sample reported higher educational levels than U.S. samples – 93 per cent of our sample had high school education versus less than 55 per cent in the United States (PHI National, 2011). We believe this is the first study to report the health status of the care aide workforce. Although it is positive that these care aides report high levels of job efficacy in the face of relatively high levels of burnout, their burnout levels are worrisome and are higher than reported levels for regulated workers (e.g., registered nurses) in our larger study (Estabrooks et al., 2012).

Burnout presents a threat to staff and has been the focus of intense research for over 35 years (Schaufeli, Leiter, & Maslach, 2009). Workplace characteristics affecting care aides – such as frequent exposure to dementia-related responsive behaviours, high workload, high acuity of residents, and little time to perform tasks for residents – mirror the environmental factors that are reported to precipitate burnout in allied health professions (Josefsson, Sonde, Winblad, & Robins Wahlin, 2007; Leiter & Maslach, 2009; Stevens, 2008). While no review has yet been published examining burnout in nursing home care aides specifically, burnout in the nursing and allied health professions presents a threat to quality of care, staff health (Kerr, Laschinger, Severin, Almost, & Shamian, 2005), and staff retention (Leiter & Maslach, 2009). Resource constraints, reductions in proportions of regulated nursing care staff, and a resident population with increasingly complex and high medical and social needs are conditions that will continue to be exacerbated as the longer-living baby boomers experience increasing rates of dementia and move through the health care, social, and residential care systems.

Although efforts are underway in most provinces to establish registries and educational strategies for the nursing home care aide workforce and to stabilize the work environment, an acute need exists to accelerate this work. Effective health human resource planning for the sector cannot proceed in Canada until we have the capacity to count these workers and are aware of their qualifications in each provincial jurisdiction. Our lack of information about the numbers and characteristics of this occupational group reduces our ability to monitor change or progress in achieving workforce improvements (Cummings et al., 2013).

From our data, we identified potential areas for improvement. We found significant provincial differences in workforce educational levels, continuing education opportunity in the workplace, and the presence of clinical education support. Each of these is modifiable and has the potential to improve quality of care for residents and quality of work life for staff in residential nursing homes. Indeed, the recent report concerning retention, recruitment, and expansion of the capacity of this workforce in the OECD countries (Fujisawa & Colombo, 2009) highlighted increasing vocational training and continuing education for non-registered health professionals working in elder care as a promising strategy for retention, recruitment, and enhancement of this workforce.

The following are the strategies, based on our data and others, that will likely have positive results for both residents of nursing homes and their caregivers if implemented:

• Mandatory registries of care aides working in residential long-term care in all provinces, and assurance that registration and training/educational requirements (both initial and ongoing) are sufficiently compatible across provinces to facilitate workforce migration across provincial boundaries.
• Processes whereby a percentage of public funding going to nursing homes is earmarked for training and ongoing education of care aides.
• Inter-provincial cooperation in developing and implementing health human resource plans – designed to ensure that we have adequate numbers of sufficiently trained care aides working with appropriate professional skill mixes in nursing homes to provide care that is not only safe, but at least meets minimum quality standards and, at best, is person-centred and an international exemplar of how care ought to be provided.
• A national discussion that addresses the complex and challenging issue of regulation of this workforce and whether it would contribute to safer and higher quality of care for nursing home residents.
• Special attention to issues of gender – this workforce is overwhelmingly female (e.g., 93% in our sample), as is the informal caregiver workforce (e.g., family members, partners) and the disproportionate and deleterious effects of long-term care on female caregivers are well-known globally (Fredriksen-Goldsen & Bonifas, 2013). However, little evidence exists in Canada or elsewhere that policy planning efforts take gender into account for workforce, service delivery, or other areas.
• Special attention to ethnicity and culture as factors in planning for workforce and service delivery needs. The majority of this workforce is non-Caucasian and multi-lingual, with English not the dominant native language. Further, the care aide workforce in most urban nursing homes is a blend of various cultural, ethnic, and linguistic groups, which is in stark contrast to the majority of baby boomers who were born between 1946 and 1964 and are of western European (predominately United Kingdom) extraction (Statistics Canada, 2013a, 2013b, 2013c).

Such an ambitious program of reform will require political, administrative, and public will, as well as cooperation across multiple sectors, actors, and groups. However, we are facing rapid increases in the aging population (with accompanying dementia) requiring residential care, with increasing complexity and dependency levels on admission. Admission occurs later in the trajectory of decline, creating much shorter lengths of stay and, consequently, higher levels of resident turnover in the system. All of these factors combine to create a pressing need for consistent and high-quality of life and quality of end-of-life care for these highly vulnerable residents. We must act urgently and with determination.

### Strengths and Limitations

While our sample is representative of urban nursing homes in the prairie provinces, we have no assurance that our findings are representative of urban nursing homes in Canada in general, and our care aide sample was not drawn randomly from a known population. We saw provincial differences suggesting that important provincial variations may exist across the country with respect to a number of characteristics. Our findings do not reflect the situation in rural Canada. Although the surveys were completed using in-person structured interviews and a rigorous quality assurance process (Squires et al., 2012), the data are self-reported and therefore subject to the challenges inherent in self-reported data.

## Conclusion

At present in Canada, we can offer only a partial and unsatisfying response to the question, “Who is looking after Mom and Dad?” We have an even sparser picture of care aide working conditions, health indicators, and work-life quality indicators – all areas that influence the quality of care. Further research will provide partial information; full information and the necessary initiatives to optimize this workforce will require action on the part of policy makers. We entrust the care of the most complex, frail, vulnerable, and challenging charges in our health and social system to this occupational group of care aides 24 hours a day – inaction is not a rational response.

## Supplementary Material

To view supplementary material for this article, please visit http://dx.doi.org/S0714980814000506

## Supplementary Material

Supplementary Material Supplementary information supplied by authors.

Supplementary Material Supplementary information supplied by authors.
