# Supplementary material for: Who is Looking After Mom and Dad? Unregulated Workers in Canadian Long-Term Care Homes
Source: Can J Aging. 2015 Mar;34(1):47–59. doi: 10.1017/S0714980814000506 (PMC4413363; doi:10.1017/S0714980814000506)
Supplement: Supplementary Material — Supplementary information supplied by authors. [file S0714980814000506sup001.doc]

Additional File 1: Description of Variables

| **Variable Class** | **Variable** | **Definition** | **Source** | **How Measured** | **How Final Score Derived** | **Alpha** |
| --- | --- | --- | --- | --- | --- | --- |
| Demographic Variables | Age | An individual’s age | TREC  Survey | Asked to indicate age according to category (e.g., < 20 years, 20-24 years….65-70 years, >70 years) | Recode to 10 year categories (< 20 years, 20-29 years, etc) | N/A |
|  | Sex | An individual’s sex | TREC  Survey | Asked for their sex:  male or female | - Male  - Female | N/A |
|  | Shift worked most of the time | Shift worked most of the time | TREC  Survey | Asked to indicate the shift they work most of the time: day, evening, or night | - Day  - Evening  - Night | N/A |
|  | Education (High school) | Education obtained | TREC  Survey | Asked if completed high school (yes/no) | - Yes  - No | N/A |
|  | Education (Care aide certificate) | Education obtained | TREC  Survey | Asked if complete HCA certificate (yes/no) | - Yes  - No | N/A |
|  | Language | First language | TREC  Survey | Asked respondent’s first language | - English  - Filipino  - Tagalog  - Other | N/A |
|  | Born in Canada | Was the care aide born in Canada | TREC  Survey | Asked if they were born in Canada (yes/no) | - Yes = born in Canada  - No = not born in Canada | N/A |
|  | Hours worked in 2 weeks | How many hours in a typical 2 weeks period is worked | TREC  Survey | Asked for number of hours worked in a typical two week period | Number of hours given = final score | N/A |
|  | Years worked as care aide | Total number of years as a care aide | TREC  Survey | Asked for number of years and months worked as a care aide | Convert the number of months to years and add it to the number of years for a total time as care aide | N/A |
|  | Years worked in Unit | Total number of years worked on unit | TREC  Survey | Asked for number of years and months worked on the unit | Convert the number of months to years and add it to the number of years for a total time on unit in years | N/A |
|  | Job Satisfaction | An individual’s perception of whether they are “satisfied” in their job (e.g. satisfied being a healthcare aide in in Unit A) | TREC  Survey | A single item scored on a 5-point likert agreement scale (Strongly Disagree to Strongly Agree) | An overall job satisfaction score is same as the original item score on 5-point likert scale | N/A |
|  | Vocational (Career) Satisfaction | An individual’s perception of whether they are “satisfied” in their career (e.g. satisfied being a healthcare aide generally) | TREC  Survey | A single item scored on a 5-point likert agreement scale (Strongly Disagree to Strongly Agree) | An overall vocational satisfaction score is same as the original item score on 5-point likert scale | N/A |
|  | Adequate Knowledge | An individual’s perception of whether they feel they have enough information to carry out their job effectively and safely | TREC  Survey | A single item scored on a 5-point likert agreement scale (Strongly Disagree to Strongly Agree) | An overall adequate knowledge score is same as the original item score on 5-point likert scale | N/A |
|  | Adequate Orientation | An individual’s perception of whether they feel they had enough orientation on the job to carry out their job effectively and safely | TREC  Survey | A single item scored on a 5-point likert agreement scale (Strongly Disagree to Strongly Agree) | An overall adequate orientation score is same as the original item score on 5-point likert scale | N/A |
|  | SF-8^TM, ‡^ |  |  |  |  |  |
|  | SF-8^TM^ (Physical) | An individual’s perception of the status of their physical and mental health over the past 4 weeks (Ware, et al, 2001) | TREC  Survey | 8 items scored on 5 or 6 point scales depending on the item | Scoring is done using a proprietary algorithm obtained when permission to use the scale is granted to produce a summary mental and physical health score (0-100%) | N/A^§^ |
|  | SF-8^TM^ (Mental) |  | TREC  Survey |  |  | N/A |
|  | Maslach Burnout Inventory (MBI) |  |  |  |  |  |
|  | MBI Exhaustion | An individual’s perception of their level of burnout. Burnout refers to a debilitating psychological condition brought about by unrelieved work stress (Maslach, 1982). Three subscales are measured: exhaustion, cynicism, efficacy | TREC  Survey | 3 items scored on a 7-point likert frequency scale (Never to Daily) for each subscale | An overall exhaustion score is derived by taking the mean of the 3 items. | .754 |
|  | MBI Cynicism |  |  |  | The overall cynicism score is derived by taking the mean of the 3 items. | .629 |
|  | MBI Efficacy |  |  |  | The overall efficacy score is derived by taking the mean of the 3 items. | .505 |
|  | Dementia-related Responsive Behaviours Towards Staff | An individual’s perception of the dementia-related responsive behaviours they experience from residents in their long-term care facility | TREC  Survey | Dementia-related responsive behaviours towards staff are measured by asking care aides to report whether or not they have experienced six kinds of behaviours by a resident in their last five shifts. A | The overall behaviour score is derived by taking a count of the kinds of dementia-related responsive behaviours that staff indicated experiencing for a total score between 0 and 6. | .720 |
| Continuing Education Variables | Attend in-services/ workshops/ courses | An individual’s perception of how frequently (in the past year) they attended in-services/ workshops/ courses | TREC  Survey | 1 item scored on a 5-point frequency scale (Strongly Never to Almost Always) | An overall score is same as the original item score on 5-point frequency scale | NA |
|  | Clinical Educator | Existence of a clinical educator in the LTC home | Facility Profile  Survey | Asked if they have educator in the facility(yes/no) | - Yes = Educator in facility  - No = No educator in facility | N/A |

^‡^ SF-8^TM^ used with permission

^§^ All 8 items contribute to deriving both the physical and mental status scales and so a separate alpha for each of the physical and mental status scales is not available. The alpha for the eight SF-8^TM^ items before derivation is .853
