# Supplementary material for: Who is Looking After Mom and Dad? Unregulated Workers in Canadian Long-Term Care Homes
Source: Can J Aging. 2015 Mar;34(1):47–59. doi: 10.1017/S0714980814000506 (PMC4413363; doi:10.1017/S0714980814000506)
Supplement: Supplementary Material — Supplementary information supplied by authors. [file S0714980814000506sup002.docx]

**Additional File 2**

**Table A. Demographic characteristics of care aide sample by owner operator model**

| **Variables** | | **Owner operator model** | | | **Total**  **(N=1381)** | **χ^2^/ANOVA** | |
| --- | --- | --- | --- | --- | --- | --- | --- |
|  |  | **Public**  **(N=408)** | **Private**  **(N=318)** | **Voluntary**  **(N=655)** |  | **P-value^*^** | **Post-hoc**^†^ |
| Age [N, (%)] | < 20 years | 3 (0.74) | 3 (0.95) | 6 (0.92) | 12 (0.87) | 0.144 | N/A |
|  | 20-29 years | 47 (11.52) | 30 (9.46) | 89 (13.59) | 166 (12.03) |  |  |
|  | 30-39 years | 80 (19.61) | 89 (28.08) | 132 (20.15) | 301 (21.81) |  |  |
|  | 40-49 years | 131 (32.11) | 99 (31.23) | 205 (31.30) | 435 (31.52) |  |  |
|  | 50-59 years | 119 (29.17) | 72 (22.71) | 167 (25.50) | 358 (25.94) |  |  |
|  | >60 years | 28 (6.86) | 24 (7.57) | 56 (8.55) | 108 (7.83) |  |  |
| Sex (N, (%)] | Male | 29 (7.13) | 19 (5.99) | 55 (8.40) | 103 (7.47) | 0.390 | N/A |
|  | Female | 378 (92.87) | 298 (94.01) | 600 (91.60) | 1276 (92.53) |  |  |
| Shift worked most of time  [N, (%)] | Day Shift | 210 (51.47) | 151 (47.48) | 304 (46.41) | 665 (48.15) | 0.474 | N/A |
|  | Evening Shift | 156 (38.24) | 128 (40.25) | 264 (40.31) | 548 (39.68) |  |  |
|  | Night Shift | 42 (10.29) | 39 (12.26) | 87 (13.28) | 168 (12.17) |  |  |
| First Language [N, (%)] | English | 197 (48.40) | 150 (47.32) | 359 (54.81) | 706 (51.20) | 0.171 | N/A |
|  | Filipino | 43 (10.57) | 37 (11.67) | 53 (8.09) | 133 (9.64) |  |  |
|  | Tagalog | 78 (19.16) | 64 (20.19) | 106 (16.18) | 248 (17.98) |  |  |
|  | Other | 89 (21.87) | 66 (20.82) | 137 (20.92) | 292 (21.17) |  |  |
| Born in Canada [N, (%)] | | 145 (35.54) | 123 (38.68) | 282 (43.05) | 550 (39.83) | 0.046 | b |
| Hours worked in 2 weeks[Mean, (SD)] | | 58.68 (17.13) | 67.53 (15.64) | 63.59 (18.31) | 63.05 (17.67) | <0.001 | a,b,c |
| Years worked as care aide [Mean, (SD)] | | 10.70 (8.54) | 10.23 (8.69) | 10.72 (9.01) | 10.60 (8.80) | 0.699 | N/A |
| Years worked in unit [Mean, (SD)] | | 4.65 (5.15) | 5.78 (6.49) | 4.56 (5.30) | 4.87 (5.58) | 0.004 | a,c |

^*^ Chi-square test for categorical variables and one-way ANOVA for quantitative variables.

^†^ The post-hoc test was examined using Bonferroni correction for continuous outcomes and (binary or multinomial) logistic regression for categorical outcomes. “a”, “b” and “c” denote the post-hoc test (multiple comparison) result for , Public-Private, Public-Voluntary and Private-Voluntary, respectively (e.g., “a” implies that there exist difference between for Public and Private).

**Table B. Demographic characteristics of care aide sample by site size**

| **Variables** | | **Site Size** | | | **Total**  **(N=1381)** | **χ^2^/ANOVA** | |
| --- | --- | --- | --- | --- | --- | --- | --- |
|  |  | **Small**  **(N=264)** | **Medium**  **(N=325)** | **Large**  **(N=792)** |  | **P-value^*^** | **Post-hoc**^†^ |
| Age [N, (%)] | < 20 years | 4 (1.52) | 4 (1.23) | 4 (0.51) | 12 (0.87) | 0.209 | N/A |
|  | 20-29 years | 40 (15.21) | 47 (14.46) | 79 (9.97) | 166 (12.03) |  |  |
|  | 30-39 years | 51 (19.39) | 64 (19.69) | 186 (23.48) | 301 (21.81) |  |  |
|  | 40-49 years | 79 (30.04) | 100 (30.77) | 256 (32.32) | 435 (31.52) |  |  |
|  | 50-59 years | 71 (27.00) | 86 (26.46) | 201 (25.38) | 358 (25.94) |  |  |
|  | >60 years | 18 (6.84) | 24 (7.38) | 66 (8.33) | 108 (7.83) |  |  |
| Sex (N, (%)] | Male | 10 (3.80) | 23 (7.10) | 70 (8.84) | 103 (7.47) | 0.026 | b |
|  | Female | 253 (96.20) | 301 (92.90) | 722 (91.16) | 1276 (92.53) |  |  |
| Shift worked most of time  [N, (%)] | Day Shift | 126 (47.73) | 159 (48.92) | 380 (47.98) | 665 (48.15) | 0.712 | N/A |
|  | Evening Shift | 111 (42.05) | 122 (37.54) | 315 (39.77) | 548 (39.68) |  |  |
|  | Night Shift | 27 (10.23) | 44 (13.54) | 97 (12.25) | 168 (12.17) |  |  |
| First Language [N, (%)] | English | 184 (69.96) | 184 (56.62) | 338 (42.73) | 706 (51.20) | <0.001 | a,b,c |
|  | Fillipino | 13 (4.94) | 34 (10.46) | 86 (10.87) | 133 (9.64) |  | a, b |
|  | Tagalog | 31 (11.79) | 47 (14.46) | 170 (21.49) | 248 (17.98) |  | a, b, c |
|  | Other | 35 (13.31) | 60 (18.46) | 197 (24.91) | 292 (21.17) |  | a, b, c |
| Born in Canada [N, (%)] | | 172 (65.15) | 159 (48.92) | 219 (27.65) | 550 (39.83) | <0.001 | a, b, c |
| Hours worked in 2 weeks[Mean, (SD)] | | 63.25 (17.29) | 62.64 (17.82) | 63.15 (17.75) | 63.05 (17.67) | 0.888 | N/A |
| Years worked as care aide [Mean, (SD)] | | 10.34 (8.26) | 10.28 (8.20) | 10.82 (9.20) | 10.60 (8.80) | 0.559 | N/A |
| Years worked in unit [Mean, (SD)] | | 5.06 (6.67) | 4.56 (4.41) | 4.93 (5.61) | 4.87 (5.58) | 0.503 | N/A |

^*^ Chi-square test for categorical variables and one-way ANOVA for quantitative variables.

^†^ The post-hoc test was examined using Bonferroni correction for continuous outcomes and (binary or multinomial) logistic regression for categorical outcomes. “a”, “b” and “c” denote the post-hoc test (multiple comparison) result for , Small-Medium Small-Large and Medium-Large, respectively (e.g., “a” implies that there exist difference between for Small and Medium).

**Table C. Comparison of work related and health outcomes among care aides by owner operator model**

| **Variables** | **Owner operator model** | | | **Total**  **(N=1381)** | **ANOVA**  **(unadjusted)** | | **ANOVA**  **(adjusted^§§^)** | |
| --- | --- | --- | --- | --- | --- | --- | --- | --- |
|  | **Public**  **(N=408)** | **Private**  **(N=318)** | **Voluntary**  **(N=655)** |  | **P-value** | **ES^††^** | **P-value** | **ES** |
| Job Satisfaction^‡^ | 4.13 (0.78) | 4.01 (0.84) | 4.08 (0.82) | 4.08 (0.81) | 0.129 | 0.003 | 0.146 | 0.033 |
| Vocational Satisfaction^‡^ | 4.23 (0.80) | 4.19 (0.72) | 4.16 (0.83) | 4.19 (0.80) | 0.353 | 0.002 | 0.479 | 0.006 |
| Adequate Knowledge^‡^ | 4.18 (0.75) | 4.07 (0.80) | 4.12 (0.74) | 4.13 (0.75) | 0.133 | 0.003 | 0.173 | 0.027 |
| Adequate Orientation^‡^ | 4.13 (0.80) | 4.03 (0.88) | 4.09 (0.79) | 4.09 (0.82) | 0.264 | 0.002 | 0.278 | 0.044 |
| Physical Health Status (0-100%)^§^ | 49.32 (7.89) | 49.13 (8.00) | 49.47 (8.07) | 49.35 (8.00) | 0.822 | 0.000 | 0.724 | 0.020 |
| Mental Health Status (0-100%)^§^ | 51.58 (8.32) | 50.85 (8.45) | 50.71 (8.98) | 51.00 (8.67) | 0.270 | 0.002 | 0.489 | 0.042 |
| MBI Exhaustion-Energy^¶^ | 2.43 (1.66) | 2.66 (1.59) | 2.48 (1.62) | 2.50 (1.62) | 0.157 | 0.003 | 0.186 | 0.006 |
| MBI Cynicism-Involvement^¶^ | 2.09 (1.52) | 2.41 (1.68) | 2.17 (1.59) | 2.20 (1.59) | 0.022 | 0.006 | 0.019 | 0.009 |
| MBI Efficacy-Inefficacy^¶^ | 5.22 (0.93) | 5.19 (0.85) | 5.24 (0.88) | 5.22 (0.88) | 0.706 | 0.001 | 0.521 | 0.024 |
| Aggression Towards Staff^**^ | 3.08 (1.68) | 3.19 (1.64) | 3.13 (1.72) | 3.13 (1.69) | 0.664 | 0.001 | 0.610 | 0.004 |

^‡^ Each variable was asked with a single item, scored on a 5-point likert scale (1-strongly disagree to 5-strongly agree).

^§^ Physical and mental health status was measured using the Health Status Short Form (SF-8™) which contains 8 items. Responses are on a five or six point scale and scoring is done using a proprietary algorithm obtained when permission to use the scale is granted. Higher scores indicate better perceived health status.

^¶^ Burnout was measured using the Maslach Burnout Inventory General Survey (MBI-GS), which consists of three subscales (emotional exhaustion, cynicism, job efficacy) each containing three items. All items are scored on a 7 point frequency like scale (0-never to 6 -daily). A mean is calculated for each sub-scale. High scores on exhaustion and cynicism with low scores on efficacy, indicate high risk for burnout.

^**^ Aggression towards staff is measured by asking HCAs to report whether or not they have experienced 6 kinds of aggression by a resident in their last 5 shifts. A count of the kinds of regression they indicated experiencing is taken for a total score between 0 and 6.

^††^Effect size (Cohen’s *f*^2^): small effect=0.02, medium effect=0.15, and large effect=0.35

^§§^Owner-operator model effect after adjusting for sex, education (HCA certificate), and born in Canada.

**Table D. Comparison of work related and health outcomes among care aides by site size**

| **Variables** | **Site size** | | | **Total**  **(N=1381)** | **ANOVA**  **(unadjusted)** | | **ANOVA**  **(adjusted^§§^)** | |
| --- | --- | --- | --- | --- | --- | --- | --- | --- |
|  | **Small**  **(N=264)** | **Medium**  **(N=325)** | **Large**  **(N=792)** |  | **P-value** | **ES^††^** | **P-value** | **ES** |
| Job Satisfaction^‡^ | 3.99 (0.90) | 4.10 (0.76) | 4.10 (0.80) | 4.08 (0.81) | 0.151 | 0.003 | 0.593 | 0.031 |
| Vocational Satisfaction^‡^ | 4.23 (0.80) | 4.18 (0.82) | 4.18 (0.79) | 4.19 (0.80) | 0.569 | 0.001 | 0.190 | 0.008 |
| Adequate Knowledge^‡^ | 4.06 (0.75) | 4.07 (0.81) | 4.17 (0.73) | 4.13 (0.75) | 0.035 | 0.005 | 0.533 | 0.025 |
| Adequate Orientation^‡^ | 3.98 (0.84) | 4.08 (0.80) | 4.12 (0.81) | 4.09 (0.82) | 0.048 | 0.004 | 0.835 | 0.043 |
| Physical Health Status (0-100%)^§^ | 48.29 (9.47) | 48.84 (7.96) | 49.91 (7.41) | 49.35 (8.00) | 0.007 | 0.007 | 0.246 | 0.021 |
| Mental Health Status (0-100%)^§^ | 50.47 (9.19) | 50.33 (8.00) | 51.45 (8.73) | 51.00 (8.67) | 0.081 | 0.004 | 0.499 | 0.042 |
| MBI Exhaustion-Energy^¶^ | 2.36 (1.67) | 2.78 (1.64) | 2.44 (1.59) | 2.50 (1.62) | 0.002 | 0.009 | 0.002 | 0.012 |
| MBI Cynicism-Involvement^¶^ | 2.03 (1.60) | 2.36 (1.64) | 2.20 (1.57) | 2.20 (1.59) | 0.042 | 0.005 | 0.043 | 0.008 |
| MBI Efficacy-Inefficacy^¶^ | 5.06 (1.01) | 5.21 (0.89) | 5.29 (0.83) | 5.22 (0.88) | 0.002 | 0.009 | 0.062 | 0.027 |
| Aggression Towards Staff^**^ | 2.75 (1.67) | 3.15 (1.63) | 3.25 (1.70) | 3.13 (1.69) | 0.000 | 0.013 | 0.001 | 0.013 |

^‡^ Each variable was asked with a single item, scored on a 5-point likert scale (1-strongly disagree to 5-strongly agree).

^§^ Physical and mental health status was measured using the Health Status Short Form (SF-8™) which contains 8 items. Responses are on a five or six point scale and scoring is done using a proprietary algorithm obtained when permission to use the scale is granted. Higher scores indicate better perceived health status.

^¶^ Burnout was measured using the Maslach Burnout Inventory General Survey (MBI-GS), which consists of three subscales (emotional exhaustion, cynicism, job efficacy) each containing three items. All items are scored on a 7 point frequency like scale (0-never to 6 -daily). A mean is calculated for each sub-scale. High scores on exhaustion and cynicism with low scores on efficacy, indicate high risk for burnout.

^**^ Aggression towards staff is measured by asking HCAs to report whether or not they have experienced 6 kinds of aggression by a resident in their last 5 shifts. A count of the kinds of regression they indicated experiencing is taken for a total score between 0 and 6.

^††^Effect size (Cohen’s *f*^2^): small effect=0.02, medium effect=0.15, and large effect=0.35

^§§^Site size effect after adjusting for sex, education (HCA certificate), and born in Canada.

**Table E. Educational opportunities for HCAs by owner operator model**

| **Individual level variable** | **Owner operator model** | | | **Total**  **(N=1381)** | **χ^2^-test**  **(p-value)** |
| --- | --- | --- | --- | --- | --- |
|  | **Public**  **(N=408)** | **Private**  **(N=318)** | **Voluntary**  **(N=655)** |  |  |
| **Education**  **[N, (%)]** |  |  |  |  |  |
| High school | 376 (92.16) | 299 (94.03) | 602 (92.33) | 1277 (92.67) | 0.569 |
| Care aide certificate | 351 (86.03) | 250 (78.62) | 551 (84.51) | 1152 (83.60) | 0.019 |
| **Attend In-services/ workshops/ courses** | 178 (43.7) | 193 (61.1) | 287 (43.9) | 658 (47.8) | <0.001 |
|  | | | | | |
| **Facility level variable** | **Owner operator model** | | | **Total (N=30)** | **Exact test^¶¶^**  **(p-value)** |
|  | **Public**  **(N=8)** | **Private**  **(N=8)** | **Voluntary**  **(N=14)** |  |  |
| Clinical Educator (Yes) | 5 (62.5) | 6 (75.0) | 11 (78.6) | 22 (73.3) | 0.866 |

^¶¶^Fisher’s exact test was used because 50% of the cells have expected count less than five.

**Table F. Educational opportunities for HCAs by site size**

| **Individual level variable** | **Site size** | | | **Total**  **(N=1381)** | **χ^2^-test**  **(p-value)** |
| --- | --- | --- | --- | --- | --- |
|  | **Small**  **(N=264)** | **Medium**  **(N=325)** | **Large**  **(N=792)** |  |  |
| **Education**  **[N, (%)]** |  |  |  |  |  |
| High school | 238 (91.19) | 299 (92.00) | 740 (93.43) | 1277 (92.67) | 0.419 |
| Care aide certificate | 197 (75.48) | 273 (84.00) | 682 (86.11) | 1152 (83.60) | <0.001 |
| **Attend In-services/ workshops/ courses** | 56 (21.3) | 148 (45.5) | 454 (57.5) | 658 (47.8) | <0.001 |
|  | | | | | |
| **Facility level variable** | **Owner operator model** | | | **Total (N=30)** | **Exact test^¶¶^**  **(p-value)** |
|  | **Small**  **(N=9)** | **Medium**  **(N=9)** | **Large**  **(N=12)** |  |  |
| Clinical Educator (Yes) | 6 (66.7) | 5 (55.6) | 11 (91.7) | 22 (73.3) | 0.180 |

^¶¶^Fisher’s exact test was used because 50% of the cells have expected count less than five.
